# Acute stress response of fathead minnows caged downstream of municipal wastewater treatment plants in the Bow River, Calgary

**DOI:** 10.1371/journal.pone.0198177

**Published:** 2018-06-21

**Authors:** Analisa Lazaro-Côté, Bastien Sadoul, Leland J. Jackson, Mathilakath M. Vijayan

**Affiliations:** Department of Biological Sciences, University of Calgary, Calgary, Alberta, Canada; Universidade de Vigo, SPAIN

## Abstract

We examined whether exposure to municipal wastewater effluent (MWWE) compromised the stress performance of laboratory-reared fathead minnows (*Pimephales promelas*) in a field setting. Adult minnows were caged at two sites upstream and three sites downstream of wastewater treatments plants (WWTPs) discharging MWWE into the Bow River, Calgary, Alberta, Canada. At each site one group of fish was sampled after a 26 day exposure to MWWE, while another group was subjected to 1-min air exposure followed by 60-min confinement and then sampled. Fish morphometrics and proximate composition were measured, and whole-body cortisol, glucose and lactate levels assessed as markers of the stress response. The whole-body protein, glycogen and lipid content were higher at the site closest to a WWTP outfall relative to the other downstream and upstream sites. There were no significant differences in whole-body cortisol levels in minnows sampled at sites either upstream or downstream of WWTPs. Acute stressor exposure significantly elevated whole-body cortisol levels in all groups, and this response was not modified by the location of the sampling sites. The whole-body metabolite profile, including glucose and lactate levels, were significantly higher in fish caged immediately downstream from WWTP inputs relative to upstream sites. There was an acute-stressor-mediated increase in whole-body lactate, but not glucose, levels and this response was independent of sampling site. The results reveal that the capacity to evoke an acute stress response was not compromised in fathead minnows caged for 26 days downstream of WWTPs in the Bow River. However, there were changes in the whole-body proximate composition and metabolite levels immediately downstream from the WWTP outfall suggesting greater accumulation of energy stores in these fish. Taken together, our results suggest that environmental factors in addition to contaminants, including higher water temperature and nutrient availability, influence the impact of MWWEs on fish stress performance.

## Introduction

Municipal wastewater effluent (MWWE) is a significant source of contamination to aquatic ecosystems, as most wastewater treatment plants are designed to primarily remove nitrogen, phosphorus, and organic matter. The final effluent consists of a complex mixture of nutrients and contaminants, including pharmaceuticals and personal care products (PPCPs), polycyclic aromatic hydrocarbons, and metals, that varies in composition depending on the waste source, treatment processes employed by each plant, and season [1–3]. PPCPs are of particular concern as many of these compounds are designed to be biologically active at low concentrations in humans, but their impact on aquatic biota in natural environments is largely unknown. Recent studies have shown that exposure to MWWE, and to the pharmaceuticals present in MWWE, impairs the adaptive cortisol stress response in fishes [4–8].

Cortisol is the primary glucocorticoid in teleosts and is used as an indicator of stress in fish [9–12]. The activation of the cortisol stress response is highly conserved among vertebrates and includes the release of corticotropin releasing factor (CRF) from the hypothalamus, which stimulates the release of adrenocorticotropic hormone (ACTH) from the corticotropic cells in the anterior pituitary gland [9,13]. ACTH released into the circulation binds to melanocortin 2 receptors (MC2R) on the interrenal cells located in the head kidney of teleost fish, and stimulates the release of cortisol [10,12,14]. The cortisol response to an acute stressor exposure is commonly used as a marker of stress performance capacity in response to contaminants in the environment [14].

In addition to the hormonal response, the subsequent metabolic responses, including changes in plasma glucose and lactate levels, are also used as markers of the secondary stress response in fish [10,12]. The primary stress hormone release plays a role in the subsequent metabolic response, as both catecholamines and cortisol are involved in glucose and glycogen regulation, as well as lactate release from the muscle [13–16]. The temporal change in the metabolic response to stressors varies, and this to a large extent is determined by the duration of exposure and/or the nutritional status of the animal [6,17–19]. Longer term exposure to contaminants, including MWWE, is energetically demanding, and may compromise the capacity of the animal to respond to additional stressors, leading to reduced fitness [4–6,18,20].

There have been several laboratory studies on the acute effects of a single contaminant using molecular and/or phenotypic endpoints in fish [21]; however, such studies do little to identify the impact of continuous exposure to complex chemical mixtures such as MWWE on natural biota. Also, most ecotoxicological studies have focused on reproductive markers of sub-lethal effects [22–24], but exposure to mixtures of contaminants is likely to affect multiple biological pathways thus necessitating a suite of biomarkers to signal early sub-lethal effects. Previous studies have clearly identified the cortisol stress axis as a target for environmental contaminants, including MWWE exposures [4,5,7,8,25]. Here we tested the hypothesis that exposure to MWWE in fathead minnows (*Pimephales promelas*), a species native to the Bow River, will compromise their cortisol response to a secondary stressor, indicative of a reduced stress performance. To test this, laboratory born and raised adult fathead minnows were caged at sites upstream and downstream of wastewater treatment plants in Calgary, Alberta, for 26 d, after which they were subjected to a secondary acute stressor *in situ*. Whole-body cortisol content was used as a marker of endocrine response to stress [26–28], while glycogen, glucose and lactate levels were used as markers of metabolic responses to MWWE exposure [4–5].

## Materials and methods

### Animals

Adult fathead minnows were obtained from the University of Lethbridge, Alberta, Canada, and maintained at the University of Calgary, Alberta, Canada at 24°C on a 14hL:10hD photoperiod. The fish were kept in 30 L tanks (Aquatic Habitats, FL, USA) with water exchange (10 L) in each tank occurring four times daily. Fish were fed daily to satiety once a day with commercial goldfish food (Nutrafin Basix, Rolf C. Hagen Inc., Montreal, QC) and bloodworms (Hikari Bio-Pure, Hayward, CA). The fish were maintained in the laboratory for three months prior to the commencement of the caging study. The animal usage and experiments were approved by the University of Calgary Animal Care Committee and were in accordance with the Canadian Council on Animal Care (CCAC) guidelines.

### Laboratory stress experiment

A preliminary laboratory study was conducted to determine the time-course of the cortisol stress-response in fathead minnows. The fish were held in 30 L tanks with air stones, and were not fed the day of the stress study. The fish were sampled either prior to or 30, 60 or 240 min after an acute stressor (1 min air exposure). Sampling consisted of quickly netting 5–6 fish (mean mass ± SEM = 0.58 ± 0.04 g) and euthanizing them by immersion in 1 g/L of MS-222 (Sigma-Aldrich, St-Louis, MO) buffered with sodium bicarbonate. The fish were weighed, and the whole body stored at -80°C for later analysis of cortisol. This experiment was repeated once.

### Field exposure and sampling

**Fig 1:**
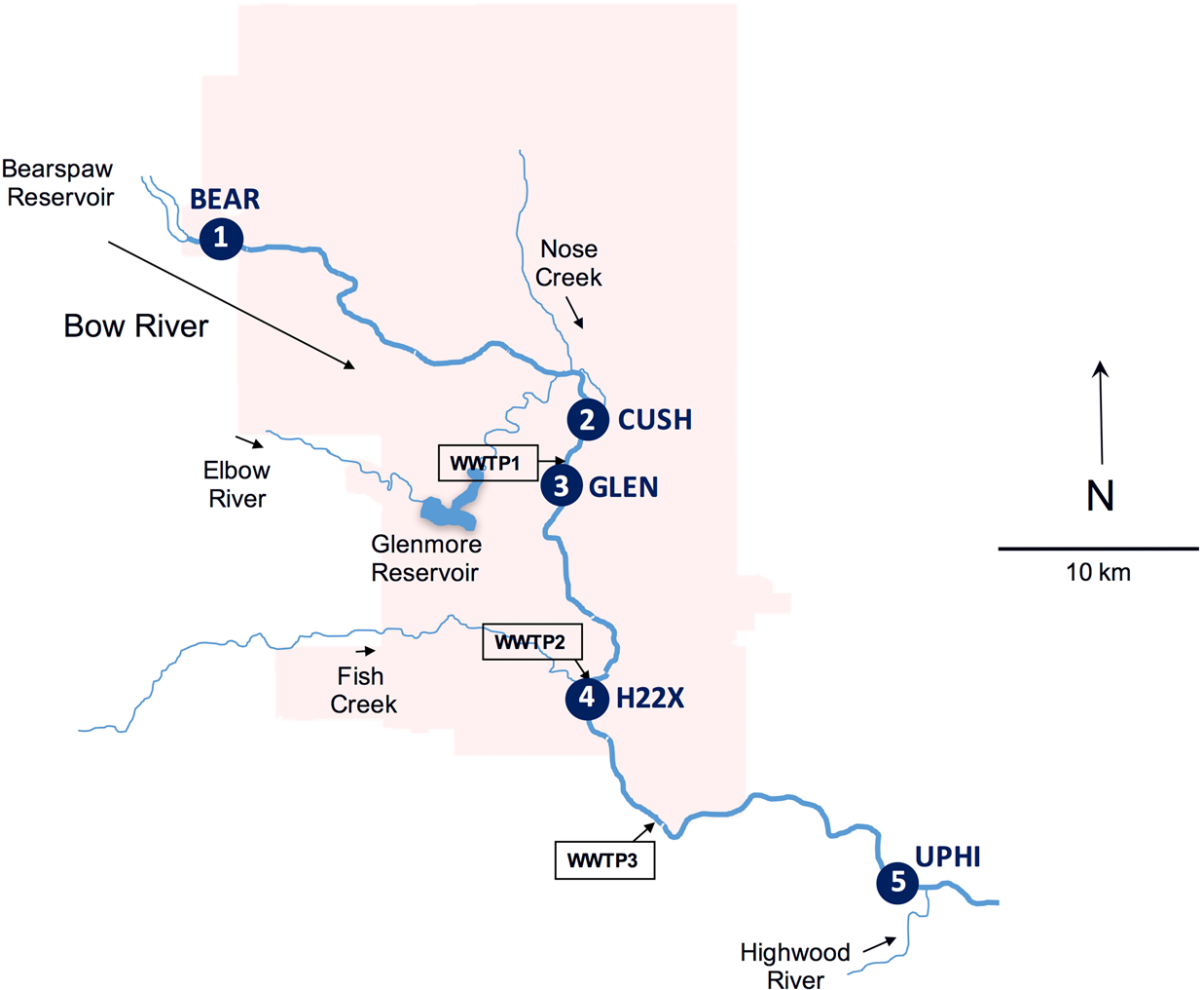
Sampling sites. Map of sampling locations along the Bow River, Alberta. Numbers represent locations where adult fathead minnows were caged relative to wastewater treatment plants (WWTP). Sampling locations consisted of two sites upstream of the City of Calgary wastewater treatment plants (BEAR: 51° 6'1.77"N, 114°16'24.18"W; CUSH: 51° 2'16.10"N, 114° 0'41.87"W), one site 15 m downstream of a WWTP side-bank discharge (GLEN: 51° 0'32.48"N, 114° 1'8.31"W), one site 250 m (H22X: 50°54'28.03"N, 114° 0'41.62"W) and one site approximately 18 km downstream from WWTP mid-channel diffusers (UPHI: 50°49'8.84"N, 113°47'31.76"W). Arrows indicate the direction of flow.

A caging study was carried out in the Bow River, Calgary, Alberta, from September to October 2016 with laboratory-reared minnows of approximately 1.41 ± 0.15 g (mean ± S.E.M) body mass. The Bow River receives discharge from three tertiary wastewater treatment plants (WWTPs) and the combined effluent flow from these plants ranged from 1–8% of the total flow of the river when fully mixed (S1 Table). Five sites were selected along the Bow River. Two sites, Bearspaw Dam (BEAR: 51° 6'1.77"N, 114°16'24.18"W) and Cushing Bridge (CUSH: 51° 2'16.10"N, 114° 0'41.87"W) were chosen upstream of the City of Calgary WWTPs (Fig 1). Of note, the site CUSH was located downstream of over 100 stormwater outfalls and a creek that receives rural and urban runoff. The other sites consisted of Glenmore (GLEN: 51° 0'32.48"N, 114° 1'8.31"W) located 15 m downstream of the WWTP1 side-bank discharge within the MWWE plume with minimal mixing with the river water, Highway 22X (H22X: 50°54'28.03"N, 114° 0'41.62"W) located 250 m downstream of the WWTP2 mid-channel diffuser, and upstream of the Highwood River (UPHI: 50°49'8.84"N, 113°47'31.76"W) which was approximately 18 km downstream of the WWTP3 mid-channel diffuser (Fig 1). Two cages consisting of galvanized steel minnow traps with the openings at each end closed with aquarium-grade sealant to cover any sharp edges [29] were installed at each site. Each cage was fitted with a 150 mm long and 100 mm diameter-wide section of PVC pipe to provide shelter for the fish. The cages were installed horizontally and held in place with two steel reinforcing bars.

Prior to caging, fish were randomly assigned to 30 L tanks to acclimate to seasonal Bow River water temperatures over a two-week period in the laboratory. The experiment consisted of randomly assigning 35–36 fish to each cage and exposing them for 26 d to the Bow River water at the appropriate sites (Fig 1). Exogenous food was not provided and the cages were cleaned every two days to avoid obstruction of water flow. Temperature, dissolved oxygen, and conductivity were measured in the river, and water samples were collected to measure pH and turbidity prior to cleaning. Survival of the fish ranged from 70 to 100% at all sites.

Fish were sampled between 9:30 am and 11:00 am following the 26 d exposure period. One subset of three to five fish per cage (six to ten fish per site) was sacrificed immediately for measuring cortisol and metabolite levels prior to stressor exposure, while another subset was subjected to an acute handling and confinement stressor consisting of one min air exposure followed by 60 min of containment in a 10 L bucket with aerators. The sampling of these fish was carried out within 5 min from the time they were removed from the cage or bucket, and consisted of euthanizing by immersion in 1 g/L of MS-222 (Sigma-Aldrich, St-Louis, MO) buffered with sodium bicarbonate. All of the fish were weighed and the fork length measured to calculate the condition factor [K = 100(body mass/fork length^3^)], followed by freezing the whole-body samples. All samples were stored in a dry-shipper (Taylor-Wharton Cryo-Express 500), then transferred to -80°C in the lab for cortisol and metabolite analyses later. The experiments were conducted in accordance with animal use protocols approved by the University of Calgary Animal Care Committee and is in accordance with the Canadian Council on Animal Care (CCAC) guidelines. The Fish Research Licence for the field sampling was issued by Alberta Environment and Parks.

### Whole-body cortisol and metabolite measurements

The whole-body samples were ground into a powder using a mortar and pestle on dry ice. A known amount of tissue was homogenized in 50 mM Tris (pH 7.5) with protease inhibitors (1:2 w/v; Roche Diagnostics, Laval, QC) using the Precellys 24 homogenizer (Bertin Technologies, Rockville, MD). For steroid analysis, an extraction of the whole-body homogenate was carried out with diethyl ether (1:5 w/v) as described previously [30]. Cortisol was quantified using an in-house competitive ELISA [30]. Perchloric acid (PCA) was added to an aliquot of the whole-body homogenate to make a 2% PCA solution, and the supernatant was used to measure glucose and glycogen levels. Whole-body glucose levels were measured by enzymatic determination with hexokinase (Sigma-Aldrich, St-Louis, MO) as described previously [31]. Whole-body glycogen levels were determined by measuring glucose before and after hydrolysis with amyloglucosidase (Sigma-Aldrich, St-Louis, MO) as described before [32–33]. Whole-body lactate levels were measured by acidifying the homogenate with a 1:2 dilution using 5% PCA, and collecting the supernatant to measure lactate enzymatically with lactate dehydrogenase (Sigma-Aldrich, St-Louis, MO) as described in Bergmeyer [34]. Protein concentration was determined in the supernatant, after spinning the homogenate (13000 x g) for 2 min, by bicinchoninic acid (BCA) method (Thermo Scientific, Rockford, IL) using bovine serum albumin as the standard (VWR, Solon, OH). Lipids were extracted from whole homogenate by a modified Folch method and measured gravimetrically [35]. All measurements were expressed per gram of wet tissue mass.

### Water PPCP measurement

Duplicate water samples from each site were collected in 500 mL amber glass bottles to measure concentrations of select PPCPs. Surrogate standards were added to the samples and extractions performed using Oasis HLB cartridges (Waters Corporation, Milford, MA) as described in Metcalfe *et al*. [36] and Garcia-Lor *et al*. [37] for the acidic and neutral PPCPs, and antidepressants, respectively. The extracts were reduced in volume under a gentle stream of nitrogen, reconstituted in 90:10 water:methanol, and isotopically labeled internal standards added. Analysis was carried out by liquid chromatography tandem mass spectrometry as described in Lajeunesse *et al*. [38] using a Poroshell 120 PFP column (2.1 x 100 mm x 2.7 μm) on an Agilent 1260 HPLC connected to a 6460 tandem mass spectrometer equipped with a Jet Stream electrospray ionization source (Agilent Technologies, Santa Clara, CA). Quantification was carried out using isotope dilution. The performance of the extraction method was verified using spike and recovery tests, and the average recoveries were 48.2% for norfluoxetine, 67.6% for fluoxetine and 81.8% to 94.0% for the remaining analytes, with percent relative standard deviation ranging from 3.0% to 12.9% for all compounds.

### Statistical analysis

All statistical analyses were conducted in R version 3.3.2 2016-10-31. Water quality parameters were analyzed with a one-way ANOVA with site as a fixed effect. Mixed-effects models were conducted with the package lme4 [39]. For the laboratory study, time post-stressor (TPS) was considered as a categorical fixed effect and experiment number (1 or 2) was used as a random factor. A Dunnett post-hoc test was performed to compare cortisol values of each time point to values prior to the stressor (TPS = 0).

For the field study, site, TPS, and the interaction between the site and TPS were included as fixed effects, while unique cage IDs were included as a random factor in the analysis of cortisol and metabolite levels. Morphometric data, as well as glycogen, protein and lipid concentrations were analyzed with site as a fixed effect and unique cage ID as a random effect. Males and females were analyzed together as there was no significant effect of sex when added to the model. Normality and homoscedasticity of the model residuals were assessed graphically. Data that did not meet the assumptions were log_10_-transformed, and the assumptions reassessed. Post-hoc comparisons were conducted using the lmerTest package [40] with a Bonferroni adjustment of the p-value to determine significant differences when significant main effects or interactions of site and TPS were detected.

## Results

### Laboratory stress study

**Fig 2:**
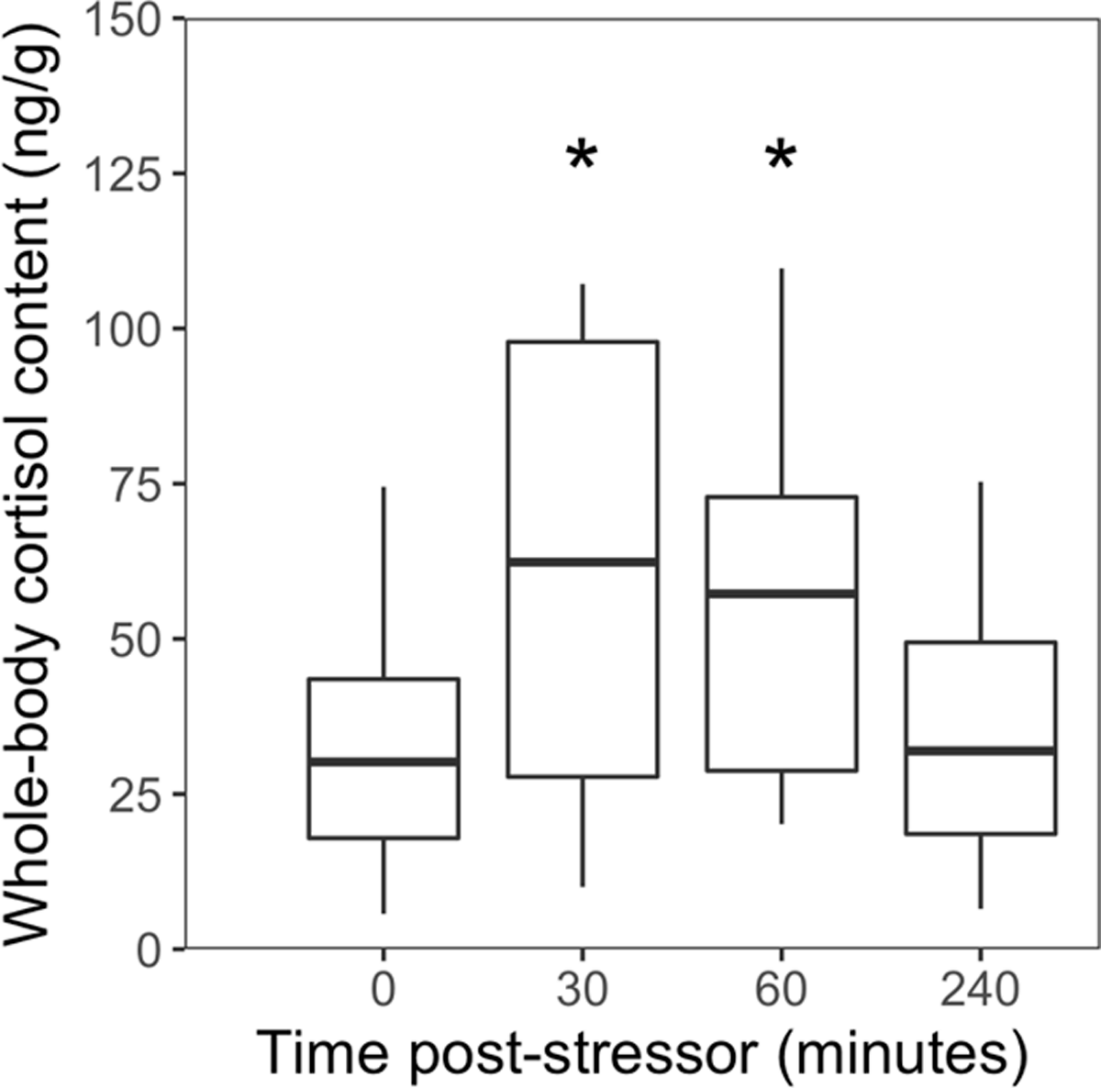
Whole-body cortisol content. Whole-body cortisol content in adult fathead minnows sampled in the lab before (0 min) and after a standardized stressor (30, 60, 240 min). For each boxplot, the line within the box represents the median whole-body cortisol concentration (n = 11–12). The box ranges from the first to the third quartile (interquartile range), the whiskers extend to ±1.5 times the interquartile range, and values beyond this are denoted as black dots. The asterisks denote a significant difference between stressed fish and unstressed fish (post-hoc Dunnett’s test; p<0.05).

There was a significant effect of time post-stressor exposure on whole-body cortisol content of fathead minnows (F_(3,41.002)_ = 3.5212, p = 0.023). Whole-body cortisol content increased significantly at 30 and 60 min following an air exposure compared to the unstressed fish, but was not significantly different at 240 min post-stressor compared to the unstressed fish (Fig 2). Based on this result, and the time required for processing the pre-stress fish tissue samples, the magnitude of cortisol response in the field was measured before and after a 60 min stressor exposure (see below).

### Bow River water quality parameters

During the September to October exposure period, the average temperature (F_(4,40)_ = 41.966, p = 8.32e-14), conductivity (F_(4,39)_ = 10697, p < 2.20e-16), dissolved oxygen concentration (F_(4,38)_ = 20.57, p = 4.35e-09), and pH (F_(4,39)_ = 31.528, p = 9.56e-12) differed significantly between sites. The average temperature and conductivity were significantly greater, while the average dissolved oxygen concentration and pH were significantly lower at GLEN compared to all other caging sites over the 26-d exposure period (Table 1). The average water temperature ranged from 12.0 to 12.5°C and dissolved oxygen concentration ranged from 9.63 to 11.04 mg/L at all sites except at GLEN, where the mean water temperature was 18.4 ± 0.2°C and mean dissolved oxygen was 6.06 ± 0.25 mg/L (Table 1). The average conductivity at all sites ranged between 298.3 and 397.4 μS/cm and the average pH between 8.00 and 8.25, except that conductivity was 2.3 to 3.1-fold greater and pH was lower by 0.77 to 1.02 at GLEN. The average turbidity at each site ranged from 3.27 to 5.06 nephelometric turbidity units (NTUs), but did not differ significantly between sites (F_(4,39)_ = 1.1804, p = 0.33; Table 1). The total ammonia-N levels were <0.05 mg/L at all sites.

**Table 1:** Water quality. Water quality parameters at each caging site along the Bow River, Calgary, Alberta from September 12 to September 30, 2016. Values represent mean ± SEM (n = 8–9).

Site	Temperature (°C)	Dissolved Oxygen (mg/L)	Conductivity (μs/cm)	pH	Turbidity (NTU)
BEAR	12.0 ± 0.3^a^	9.63 ± 0.51^b^	298.3 ± 1.5^a^	8.25 ± 0.03^b^	4.28 ± 1.37
CUSH	12.0 ± 0.5^a^	10.45 ± 0.14^b^	325.8 ± 2.1^b^	8.18 ± 0.08^b^	3.27 ± 0.36
GLEN	18.4 ± 0.2^b^	6.06 ± 0.25^a^	928.5 ± 3.6^e^	7.23 ± 0.08^a^	4.32 ± 0.53
H22X	12.5 ± 0.4^a^	11.04 ± 0.56^b^	376.0 ± 2.8^c^	8.19 ± 0.09^b^	5.06 ± 0.60
UPHI	12.3 ± 0.3^a^	9.91 ± 0.53^b^	397.4 ± 2.3^d^	8.00 ± 0.08^b^	4.33 ± 1.14

^a^ Letters that are different within columns represent statistically significant differences between sites following multiple comparisons with Bonferroni’s adjusted p-value.

As a qualitative assessment, we measured select PPCPs in a single water sample collected from each site. Both upstream sites showed non-detectable levels of PPCPs, with the exception of caffeine at the CUSH site (Table 2). The concentration of PPCPs immediately downstream of WWTP (GLEN) was similar to that seen in the undiluted MWWE (collected from WWTP3 and this data is only provided for comparison), but the farther away from the WWTP, at UPHI and H22X, the PPCPs were diluted to <15% of the concentration seen at GLEN (Table 2).

**Table 2:** Contaminant levels. Select pharmaceuticals and personal care products (PPCPs) in μg/L measured at each caging site along the Bow River in September 2016.

Compound	MDL	BEAR 12/09	CUSH 13/09	GLEN 12/09	H22X 12/09	UPHI 13/09	WWTP3* 12/09
*Neutral PPCPs*							
Cotinine	0.005	ND	ND	0.034	0.005	0.005	0.034
Caffeine	0.010	ND	0.010	0.127	0.021	0.023	0.022
Trimethoprim	0.004	ND	ND	0.227	0.010	0.018	0.227
Carbamazepine	0.002	ND	ND	0.393	0.017	0.034	0.482
*Acidic PPCPs*							
Naproxen	0.004	ND	ND	0.086	0.004	0.009	0.029
Diclofenac	0.004	ND	ND	0.480	0.031	0.071	0.507
Ibuprofen	0.007	ND	ND	0.060	ND	ND	ND
Gemfibrozil	0.007	ND	ND	0.017	ND	ND	ND
*Antidepressants*							
Fluoxetine	n/a	ND	ND	0.002	ND	ND	0.005
Norfluoxetine	n/a	ND	ND	0.014	ND	ND	0.013
Venlafaxine	n/a	ND	ND	0.472	0.015	0.031	0.538
O-Desmethylvenlafaxine	n/a	ND	ND	1.514	0.054	0.110	1.735

MDL, method detection limit; ND, not detected

* We did not have undiluted MWWE from WWTP1, so the undiluted sample from WWTP3 is provided for comparison.

### Fish morphometrics

The fish mass (F_(4,156)_ = 9.279, p = 9.42e-07), fork length (F_(4,159)_ = 6.2101, p = 0.00011), and condition factor (CF; F_(4,155)_ = 12.281, p = 1.06e-08) differed significantly between sites. The mass and length of fathead minnows were significantly higher at GLEN compared to all other sites following the exposure period (Table 3). There were no other significant differences in mass or length between fish at other sites (Table 3). The CF was significantly higher at GLEN compared to BEAR and UPHI, while fathead minnows at CUSH had significantly higher CF compared to all other sites except GLEN (Table 3).

**Table 3:** Fish morphometrics. Length (mm), weight (g) and condition factor of adult fathead minnows caged at each site along the Bow River, Calgary, Alberta. Values shown as mean ± SEM (n = 26–39).

Site	Length (mm)	Weight (g)	Condition factor
BEAR	46 ± 1^a^	1.11 ± 0.09^a^	1.043 ± 0.017^a^
CUSH	46 ± 1^a^	1.28 ± 0.08^a^	1.233 ± 0.039^c^
GLEN	51 ± 1^b^	1.63 ± 0.08^b^	1.200 ± 0.017^bc^
H22X	46 ± 1^a^	1.14 ± 0.08^a^	1.103 ± 0.018^a^^b^
UPHI	45 ± 1^a^	0.99 ± 0.07^a^	1.061 ± 0.023^a^

^a^Letters that are different within columns represent statistically significant differences between sites following multiple comparisons with Bonferroni’s adjusted p-value.

### Fish proximate composition

**Fig 3:**
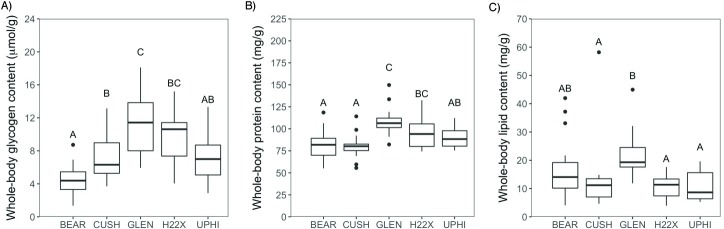
Proximate composition. Whole-body glycogen (A; n = 14–20) protein (B; n = 14–20) and lipid (C; n = 10–18) content in adult fathead minnows caged in the Bow River, Calgary, Alberta. For each boxplot, the line within the box represents the median whole-body concentration. The box ranges from the first to the third quartile (interquartile range), the whiskers extend to ±1.5 times the interquartile range, and values beyond this are denoted as black dots. Different letters indicate significant differences between sites (p<0.05).

Following the 26-d exposure, there were significant differences in whole-body glycogen (F_(4,82)_ = 15.069, p = 2.83e-09), protein (F_(4,84)_ = 12.02, p = 9.05e-08), and lipid content (F_(4,65)_ = 5.4646, p = 0.0007431) between the sites. Whole-body glycogen content was significantly higher in fish caged at GLEN compared to all sites, except the downstream H22X site (Fig 3A). The fish in the upstream site CUSH, and the downstream site H22X, had significantly higher glycogen content compared to the upstream BEAR site (Fig 3A). There were no significant differences in whole-body glycogen levels between fish from CUSH, H22X, and UPHI (Fig 3A). Whole-body protein content in fathead minnows was significantly higher in fish from GLEN compared to all other sites except H22X (Fig 3B). Protein levels at H22X were significantly higher than the upstream sites, while there were no significant differences in protein content between any of the other sites (Fig 3B). Total lipid content in the minnows was significantly higher at GLEN compared to all other sites, except the upstream site BEAR (Fig 3C). The total lipid content was not significantly different in the minnows in any of the other sites (Fig 3C).

### Whole-body cortisol and metabolite levels

**Fig 4:**
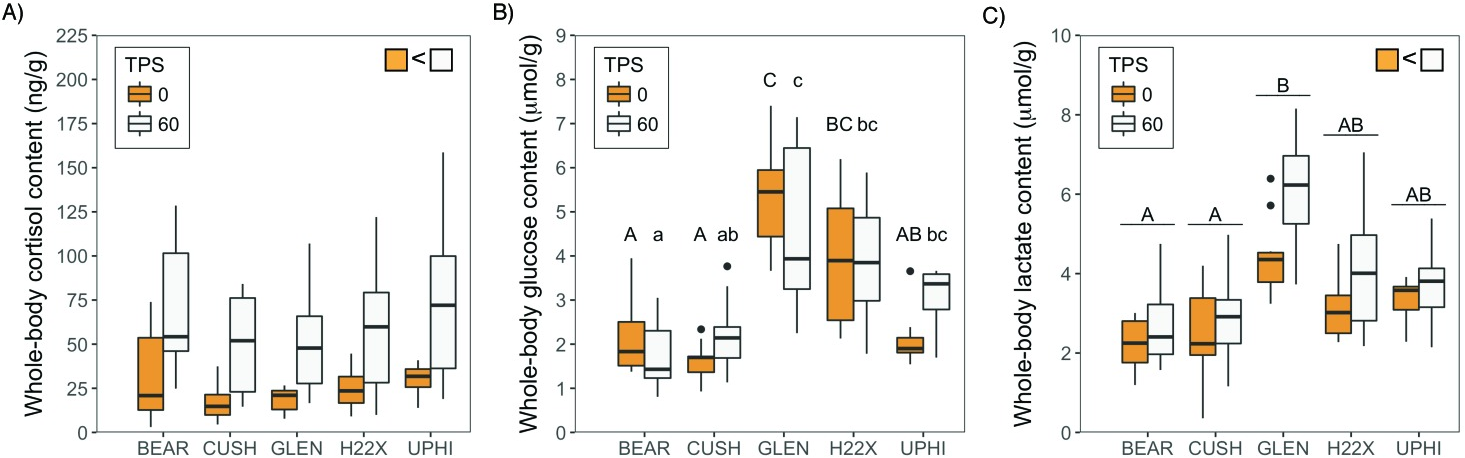
Stress response. Whole-body cortisol (A), glucose (B), and lactate (C) content in adult fathead minnows caged at five sites along the Bow River, Alberta, from September to October 2016, prior to (0 min; orange boxplots) and after a standardized stressor (60 min; white boxplots). For each boxplot, the line within the box represents the median whole-body concentration (n = 7–10). The box ranges from the first to the third quartile (interquartile range), the whiskers extend to ±1.5 times the interquartile range, and values beyond this are denoted as black dots. Different letters indicate significant differences between sites (p<0.05). The inset indicates a significant main effect of time sampled post-stressor (TPS; p<0.05).

Whole-body cortisol levels in fathead minnows exposed to varying dilutions of MWWE for 26 d were not significantly different between sites (F_(4,5.286)_ = 0.787, p = 0.58; Fig 4A). The acute secondary stressor significantly elevated whole-body cortisol levels compared to the pre-stressor levels regardless of the site sampled (F_(1,74.439)_ = 40.400, p = 1.49e-08; Fig 4A). There was no significant interaction effect of sampling site and time sampled post-stressor on whole-body cortisol in the present study (F_(4,74.430)_ = 0.329, p = 0.86; Fig 4A). There was a significant interaction effect of sampling site and time sampled post-stressor on whole-body glucose levels (F_(4,77)_ = 2.5148, p = 0.048; Fig 4B). Whole-body glucose levels were not significantly different in fish sampled 60 min following stressor exposure compared to unstressed fish at any site, but glucose levels of fish differed between sites for unstressed and stressed fish (Fig 4B). In unstressed fish, whole-body glucose levels were significantly higher in fish at the GLEN and H22X sites compared to the upstream sites (Fig 4B). The downstream UPHI site had significantly lower whole-body glucose levels compared to the GLEN site, while there were no significant differences between either of the upstream sites, and between UPHI and the upstream sites (Fig 4B). The stressed fish also showed site differences in glucose levels similar to that seen in the unstressed fish. The only exception was that the whole-body glucose level in fish sampled at CUSH was not significantly different from H22X (Fig 4B). Whole-body lactate levels differed significantly between sites (F_(4,4.840)_ = 13.6364, p = 0.0075). Fathead minnow sampled from the GLEN site had significantly greater whole-body lactate levels compared to the upstream sites, but not the downstream sites (Fig 4C). Fish sampled following the application of the acute stressor had significantly elevated lactate levels compared to unstressed fish (F_(1,74.129)_ = 10.5578, p = 0.0017; Fig 4C). There was no significant interaction effect of sampling site and time sampled post-stressor on whole-body lactate in the present study (F_(4,74.116)_ = 1.1499, p = 0.34; Fig 4C).

## Discussion

Our results demonstrate a robust cortisol response to an acute stressor exposure in fathead minnows caged downstream of the WWTPs in the Bow River, Calgary. The perception of a stressor, including an acute physical stressor, activates the HPI axis to stimulate the synthesis and release of cortisol into the circulation [9,10,12]. This was also the case in fathead minnows as the whole-body cortisol levels were elevated by one hour after a stressor exposure compared to the pre-stress levels in a laboratory setting. Similar increases in whole-body cortisol levels after an acute stressor exposure has also been reported in zebrafish [30]. This capacity to evoke an acute cortisol response to an acute stressor in the fathead minnows was not impacted by a 26 d exposure to different dilutions of MWWE in the Bow River. The capacity of fish to elicit an acute cortisol response to a stressor has been used as a biomarker for adrenotoxicants in the aquatic environment [14]. Indeed, chronic exposure to contaminants, including PCBs and metals, inhibit the acute activation of the HPI axis in fish [14]. Also, studies have shown that exposure to either MWWE [4,5,7,8] or pharmaceuticals present in MWWE [41,42] inhibit the cortisol stress axis activity in fishes. Considering this, the absence of a HPI axis dysfunction seen in the present study suggests either that the MWWE inputs into the Bow River are devoid of adrenotoxicants or that they are at concentrations low enough to not disrupt the HPI axis functioning in fathead minnows. As it is well established that adrenotoxicants are present in the MWWEs [1–3,14], the latter is more likely, and the endocrine response to stress may also be influenced by other factors, including the water quality, life stage, and the sensitivity of the fish species to contaminants.

In the present study, the downstream site GLEN, which was only within 15 m of WWTP1, had surface levels of measured PPCPs similar to that seen in the undiluted MWWE collected from WWTP3 (Table 2). The finding that there was no effect on the cortisol stress response in fathead minnows compared to our previous study with juvenile trout in the Speed River, Ontario [4,5] may be due to several factors, including the distinctive contaminant profiles and concentrations of the MWWEs, differences in the hydrology and water quality of the receiving environment, and the species and the life stage of the animal tested. For instance, the chemical composition of MWWEs and the concentrations reported in surface waters can vary considerably due to several factors, including daily and seasonal differences in the MWWE, wastewater treatment type, and influent source [1,2]. The WWTPs discharging into the Bow River during the exposure period were estimated to contribute 1–8% to the river flow when fully mixed (S1 Table), while the WWTP discharging into the Speed River has been estimated to constitute 44% of the river flow [43]. Consequently, PPCP concentrations in the Bow River at the exposure sites may be lower relative to the concentrations experienced by caged trout in the Speed River [4,5]. Furthermore, differences in species or life stage sensitivities to toxicant exposure may also have played a role in the observed differences. For instance, fathead minnows are less sensitive to certain toxicants compared to rainbow trout as determined by 96h LC50 bioassays [44,45]. Although the species and life stage sensitivities to cortisol stress axis activity due to contaminants have not been tested, we cannot rule out the possibility that the adult fathead minnows may be less sensitive to adrenotoxicants in MWWEs compared to juvenile rainbow trout [4,5]. Regardless of the factor(s) affecting stress axis activation, our results highlight the possibility that certain receiving environments for MWWE may not compromise the capacity to elicit a cortisol stress response in fishes. Comparing the PPCP concentrations in the MWWEs at a point in time to those from other WWTPs is not appropriate given the inherent variability in their effluent composition, either due to the treatment methods and/or dilution of the effluent due to the hydrology of the receiving environment, as well as the differing water quality parameters. Our results reveal that the acute stress-mediated cortisol levels of adult fathead minnow were not compromised, leading to the proposal that water quality characteristics and nutrient availability may modulate the impact of adrenotoxicants in the Bow River, especially given that the cages at GLEN were directly in the plume of MWWE from WWTP1.

Although, we did not see an impact on the cortisol stress axis response, there were clear changes in the metabolic status of the fish downstream of the WWTPs. For instance, whole-body glucose levels were greater in fish caged downstream from WWTPs (GLEN and H22X) compared to upstream sites, and lactate levels were significantly greater at GLEN compared to the upstream site BEAR, suggesting higher metabolic capacity of fish in these downstream sites. The whole-body lactate level is indicative of glycogen breakdown and anaerobic metabolism, which is normally associated with higher activity of fishes [17,46]. Therefore, the higher glucose and lactate levels point to a combination of enhanced energy substrate availability, as well as increased muscle activity in fish caged at GLEN. One striking observation was the greater water temperature at GLEN compared to the other sites (Table 1), which may have played a key role in the activity level and metabolic changes seen in the caged fish. The metabolic rate of fathead minnows increases with temperature [47], and the maximum metabolic rate reported was at a temperature of 28°C [48]. Moreover, there may be greater food availability, as previous studies in the Bow River have shown increased primary producer biomass [49], and invertebrate abundance [50] associated with increased nutrient enrichment downstream from WWTPs. This is supported by the larger body mass of minnows at GLEN compared to all other caging sites. Also, the greater whole-body protein, lipid and carbohydrate composition in fish caged at GLEN relative to fish sampled from upstream sites supports a positive energy balance for growth at this downstream MWWE site. Although there was also a higher whole-body protein, glycogen, and glucose content in fish at H22X, a downstream site farther than GLEN, compared to the reference site (BEAR), there were no changes in body mass in fish at that site or the site farthest from WWTPs (UPHI). This also coincides with lower contaminant concentrations compared to GLEN (Table 2) at these two sites, and the water temperatures were similar to those of the upstream sites, pointing to a loss of any metabolic effects due to MWWE 18 km downstream of WWTP (UPHI), which is downstream from Calgary's urban footprint. Together, these results lead us to propose that the higher temperature at GLEN, in addition to the contaminants, may have played a prominent role in the higher nutritional status and altered energy metabolism in those fish. Whether the higher nutritional status of the fish at GLEN may have masked the effect of adrenotoxicants remains to be determined.

The secondary responses to stressors, including whole-body glucose and lactate levels were not impacted downstream of WWTPs. The increase in stress hormones mediates the mobilization of energy substrates to allow an animal to regain homeostasis following an acute stressor [51]. Indeed, studies have shown that the release of cortisol following an acute stressor stimulates glycogenolysis and gluconeogenesis, thereby increasing circulating levels of free-glucose [13,17,20,41]. Studies have also found that a stressor resulting in physical exercise increases glycogen breakdown in the muscles therefore releasing lactate into the muscles and into the circulation [46]. Measurements of glucose one hour following an acute stressor did not result in significant changes in glucose levels in the present study. Increases in plasma concentrations of glucose in response to stressors are variable and the peak levels range anywhere from half hour to several hours post-stressor, and may be dependent on the species, type and intensity of the stressor, and the nutritional status of the animal [5,17,33,52]. As we only had a one hour time point post-stressor we may have missed a more rapid stressor-mediated change in glucose levels in fathead minnows. Also, due to the small size of the adult fathead minnows, whole-body rather than plasma samples were analyzed in this study, which may have masked changes in glucose levels in response to the acute stressor [30]. However, lactate levels were higher after the stressor exposure clearly indicating increased muscle metabolism, as lactate production is indicative of anaerobic metabolism [17,46]. The elevation of lactate levels, regardless of site effects, suggests that the metabolic capacity to cope with a stressor may also not be compromised in adult fathead minnows caged downstream from WWTPs in the Bow River.

A notable finding from this study was a higher whole-body glycogen content in fish caged at CUSH, a site upstream from Calgary's WWTPs, but downstream of over 100 stormwater outfalls and a creek that receives rural and urban runoff, compared to BEAR. The glycogen content at CUSH was comparable to fish caged at downstream sites suggesting input of contaminants to the upstream site as the water quality parameters were similar between the upstream and downstream sites, except for the higher water temperature at GLEN. The stormwater drains collect urban runoff containing polycyclic aromatic hydrocarbons, nutrients, sediments, and metals [53]. During precipitation events, stormwater is released into the nearest water-body either without treatment or may be collected in stormwater retention ponds, which rely primarily on sedimentation for the removal of contaminants [54]. Therefore, it is essential that a reference site used for MWWE monitoring be carefully chosen by taking into account the contribution from other extraneous sources, including stormwater outfalls, to water quality upstream of WWTP.

In conclusion, adult fathead minnows caged downstream of WWTPs in the Bow River elicited a cortisol response to a standardized acute stressor exposure. Although the intermediary metabolism and proximate composition of fish were impacted at the site closest to the WWTP, the effects may not be fully attributable to the contaminants in the MWWE. Additional environmental factors, including temperature and input of contaminants and nutrients from sources other than WWTPs may have contributed to the observed metabolic effects in caged fathead minnows. Whether the increase in energy substrate levels in fish sampled downstream of stormwater and WWTPs impact the fitness of individuals remains to be determined. If the fish are indeed feeding more downstream of WWTPs, lipophilic contaminants may bioaccumulate in tissue which may enhance the metabolic demand of the animal and/or lead to long-term impacts on their performances [55]. The longer-term effects of MWWE exposure on animal performances and fitness remains to be evaluated. It is important to highlight that the complexity of the caging environment, either directly or indirectly mediated by contaminants and/or nutrients from the WWTPs or other sources, may modify the MWWE-mediated effects on fish. While it is difficult to discern the direct effects of MWWE under such circumstances, a complementary lab-based or controlled study in a natural setting using the MWWE at different dilutions may shed light on the potential of the chemical cocktail in affecting the endocrine function and metabolism of fish.

## Supporting information

S1 Table Estimated municipal wastewater effluent flow contribution into the Bow River. Mean daily flow from each of Calgary’s wastewater treatment plants, and the Bow River in m^3^/s, and estimated effluent flow contribution into the Bow River (% effluent) during the 26 d exposure period in September and October 2016.
(PDF)

S2 Table Complete dataset. (XLSX)
