# Supplementary material for: Acute stress response of fathead minnows caged downstream of municipal wastewater treatment plants in the Bow River, Calgary
Source: PLoS One. 2018 Jun 21;13(6):e0198177. doi: 10.1371/journal.pone.0198177 (PMC6013188; doi:10.1371/journal.pone.0198177)
Supplement: S1 Table — Mean daily flow from each of Calgary’s wastewater treatment plants, and the Bow River in m3/s, and estimated effluent flow contribution into the Bow River (% effluent) during the 26 d exposure period in September and October 2016. (PDF) [file pone.0198177.s001.pdf]

Supporting information

S1 Table. Estimated municipal wastewater effluent flow contribution into the Bow River. Mean daily flow from each of Calgary's wastewater treatment plants, and the Bow River in m^3/s, and estimated effluent flow contribution into the Bow River (% effluent) during the 26 d exposure period in September and October 2016.

WWTP	Exposure period	Bow River flow (m^3/s)	WWTP flow (m^3/s)	Effluent contribution (%)
1	Sep. 11 – Oct. 7	54.3	4.5	8.3
2	Sep. 8 – Oct. 4	58.5	0.4	0.6
3	Sep. 7 – Oct. 3	58.5	1.1	1.9
